# Endodontic Treatment of a Maxillary First Molar With Two Separate Palatal Roots: A Case Report

**DOI:** 10.7759/cureus.51907

**Published:** 2024-01-08

**Authors:** He Liu, Ya Shen

**Affiliations:** 1 Division of Endodontics, Department of Oral Biological & Medical Sciences, Faculty of Dentistry, University of British Columbia, Vancouver, CAN; 2 Division of Endodontics, Department of Oral Biological & Medical Sciences, Faculty of Dentistry, University of British Columbia, Vacnouver, CAN

**Keywords:** dental operating microscope, extra palatal root, cone- beam computed tomography, maxillary first molar, root canal treatment

## Abstract

Maxillary first molars exhibit considerable anatomical variation, with a single palatal root being the most common occurrence, while two palatal roots are notably less frequent. This case report details the endodontic treatment of a maxillary first molar with two separate palatal roots. It highlights the critical importance of recognizing these anatomical variations and their unique challenges during endodontic procedures. This report emphasizes the essential role of advanced diagnostic methods, such as cone-beam computed tomography, and the use of microscopic techniques in identifying and treating such cases.

## Introduction

The primary goal of endodontic treatment is to eliminate bacteria from the root canal system to prevent reinfection, promote the healing of periapical tissues, and ensure the long-term functionality of the tooth [1-3]. This objective is achieved by meticulously cleaning and shaping the root canals to remove diseased tissue, bacteria, and their byproducts [4-6]. Following this, the canals are sealed with an inert filling material to avert future microbial ingress [7]. 

The anatomical intricacies of maxillary molars pose significant challenges in endodontic treatment, often necessitating sophisticated diagnostic and therapeutic strategies [8-10]. While most maxillary molars typically have three roots - two buccal and one palatal - there have been rare instances of maxillary molars with double palatal roots, with a reported global incidence of 0.047% [10-14]. This anatomical variability underscores the need for clinicians to understand these structural characteristics in-depth for effective endodontic treatment [10].

Christie et al. explored endodontically treated or extracted maxillary molars, identifying three unique radicular configurations based on root shape and separation [12]. They categorized these into three types [12]. Type I consists of maxillary molars with two divergent, often lengthy, and tortuous palatal roots and buccal roots that are less divergent and resemble a "cow horn". These teeth display four distinct root apices on radiographs. Type II has four separate roots that are shorter, run parallel, and possess distinct buccal and lingual root morphologies with blunt apices. This type might appear to have just mesial and distal roots on buccolingual superimposition radiographs. Type III features constricted root morphology, with mesiobuccal, mesiopalatal, and distopalatal canals surrounded by a dense web of root dentin and a distinct, possibly diverging distobuccal root.

Successfully treating maxillary molars, especially those with double palatal roots, depends on accurately identifying and managing all root canals [2,15,16]. The challenge lies not only in the number of roots but also in their varied shapes and trajectories, with some exhibiting extreme curvatures that complicate treatment [16]. Recognizing these anatomical variations is crucial, as missed canals or improper treatment can fail [2,15-17]. A comprehensive understanding of the root canal system in these teeth is, therefore, essential for effective treatment planning and execution. Recent advances in diagnostic imaging, particularly cone-beam computed tomography (CBCT), have transformed the diagnosis and treatment of complex root canal systems [18]. CBCT provides a three-dimensional view of the tooth, offering critical insights into root canal number, location, and path [18]. Moreover, the development of microsurgical endodontic techniques and instruments has greatly enhanced endodontic treatments' precision and success rates [16].

This case report aims to underscore the challenges in treating a maxillary first molar with two separate palatal roots, highlighting the necessity of incorporating advanced diagnostic and treatment techniques in contemporary endodontic practice.

## Case presentation

A 26-year-old male Chinese patient presented to the Department of Endodontics with long-term sensitivity to thermal stimuli and a moderate level of spontaneous pain, rated 4 on the visual analog scale, in the upper right posterior teeth for the past month. Exposure to hot or cold drinks and activities such as chewing or biting did not exacerbate the pain. The patient did not use any antibiotic or painkiller medication for pain relief. Furthermore, the pain was not severe enough to disturb his sleep or wake him up. His medical history was non-contributory. The patient's general health condition was good, classified as ASA I, with no signs and symptoms of any systemic diseases. The patient maintained good oral hygiene status and did not have any history of deleterious and/or parafunctional habits. The patient's upper left first molar had previously received root canal therapy; however, the exact timing and additional treatment details are unavailable.

Clinical examination revealed normal gingiva around the upper right first molar, negative palpation at the root apex, extensive caries on the distal occlusal surface, normal tooth mobility, and negative percussion test. Periodontal probing results were within normal limits. The upper right first molar and healthy control teeth (upper right second premolar, lower right first molar, and lower left first molar) were subjected to a cold test using Endo-Frost cold spray (Roeko, Coltène/Whaledent Inc., Langenau, Germany). The response in control teeth was characteristic of a healthy pulp: a brief, sharp pain that subsided almost immediately after removing the stimulus, indicating normal pulp vitality. In contrast, the upper right first molar exhibited a prolonged and lingering pain that continued for thirty seconds after the cold stimulus was removed. The gingiva around the upper left first molar was healthy. An amalgam restoration was visible on the occlusal surface, with no secondary caries at the margins. The tooth was stable with normal mobility, and palpation and percussion tests yielded negative results. Periodontal probing results were within normal limits. The upper right first molar had a sixth cusp near the mesial side of Carabelli's cusp. In contrast, the opposing upper left first molar only had Carabelli's cusp and did not exhibit a sixth cusp.

**Figure 1:**
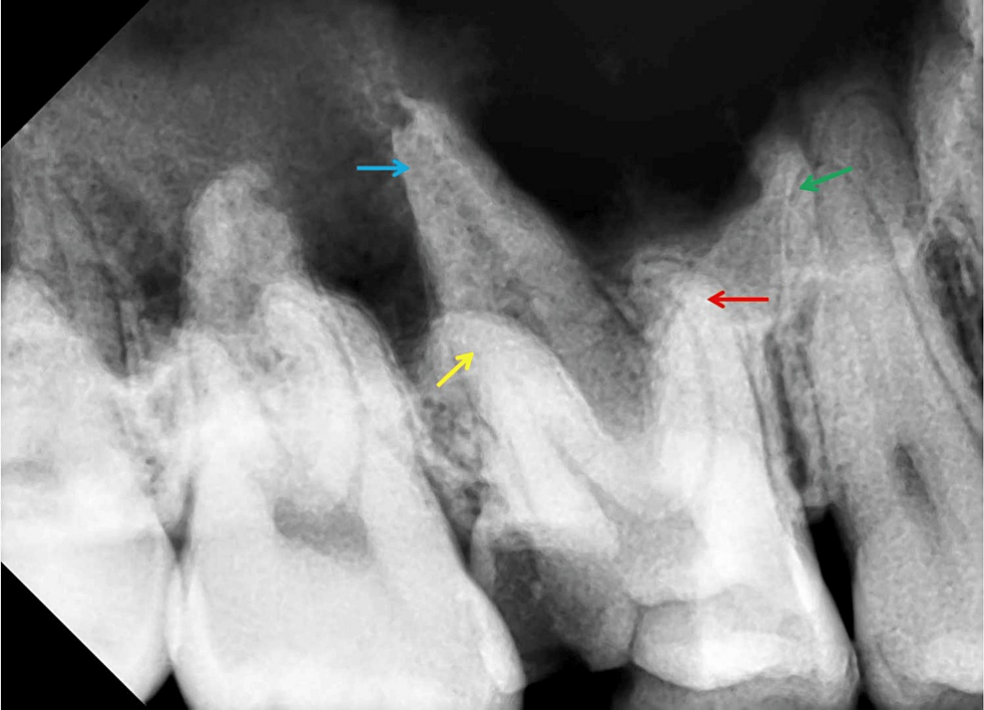
The preoperative periapical radiograph of the upper right first molar. The tooth has four roots, indicated as follows: the mesiobuccal root (marked by a red arrow), the distobuccal root (yellow arrow), the mesiopalatal root (green arrow), and the distopalatal root (blue arrow). Notably, the palatal roots are widely spaced. The apices of the mesiobuccal, distobuccal, and mesiopalatal roots exhibit a distal curvature, collectively resembling the shape of cow horns.

A periapical radiograph showed distal occlusal decay and possible pulp involvement in the upper right first molar, with no apparent periapical radiolucency and a suspected double palatal root (Figure 1).

**Figure 2:**
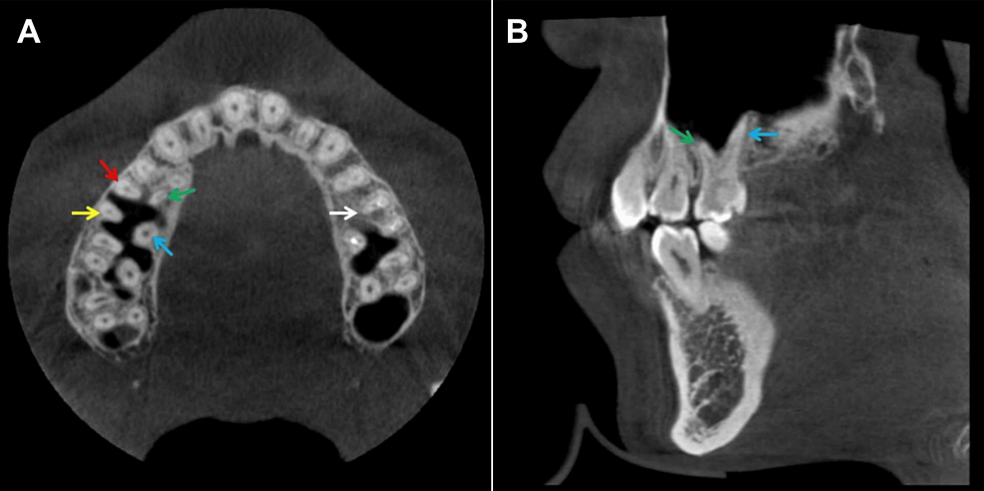
The cross-sectional (A) and sagittal (B) views of cone-beam computed tomography (CBCT). The cross-sectional view (A) of CBCT reveals that the upper right first molar has four roots, indicated as follows: the mesiobuccal root (marked by a red arrow), the distobuccal root (yellow arrow), the mesiopalatal root (green arrow), and the distopalatal root (blue arrow). In contrast, the upper left first molar (white arrow) has two buccal roots and a single palatal root. The sagittal view (B) of CBCT shows that the mesiopalatal root (green arrow) and the distopalatal root (blue arrow) of the upper right first molar are widely spaced apart.

CBCT confirmed four roots in the upper right first molar, including the double palatal roots, while the upper left first molar had three roots with a single palatal root (Figure 2A). The CBCT also revealed that the double palatal roots of the upper right first molar were widely spaced (Figure 2B).

The diagnosis was symptomatic irreversible pulpitis with normal periapical tissue in the upper right first molar. The treatment plan included root canal treatment followed by full crown restoration for the upper right first molar. The patient was informed and consented to the treatment plan and procedures throughout the pre-operative and entire treatment process. 

**Figure 3:**
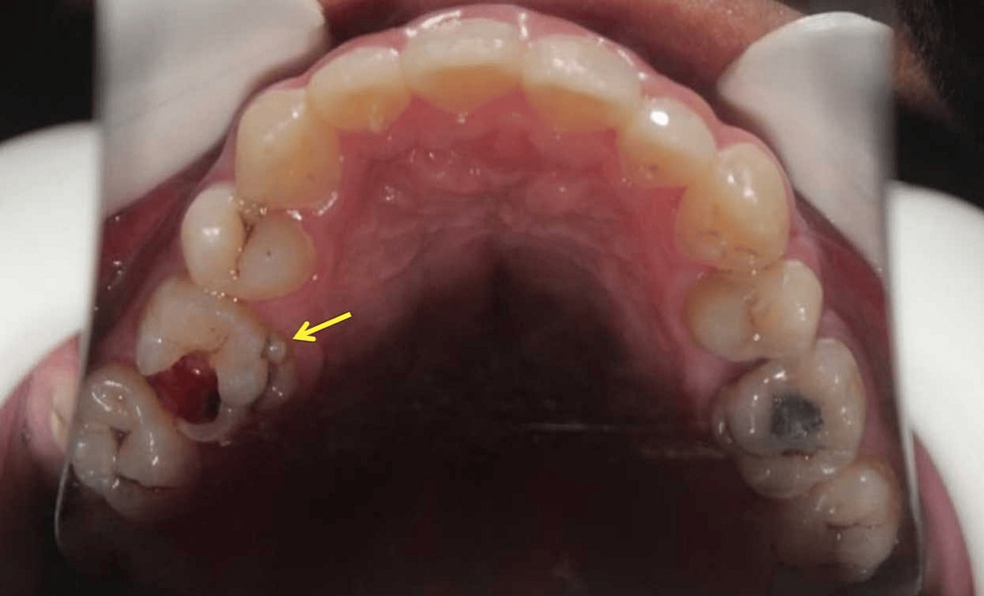
The intraoral photograph. The upper right first molar has a sixth cusp near the mesial side of Carabelli's cusp (indicated by the yellow arrow). In contrast, the opposing upper left first molar only has Carabelli's cusp and does not exhibit a sixth cusp.

Following the administration of local infiltration anesthesia, a full capsule (1.7mL) of 4% articaine with 1:100,000 adrenaline (Septanest, Septodont, France) was injected on the buccal side, and a half capsule (0.85mL) was administered on the palatal side. Subsequently, decay removal and access opening procedures were carried out under a dental operating microscope (OPMI PICO, Carl Zeiss, Oberkochen, Germany). An intraoral photograph was taken (Figure 3), and the tooth was isolated with a rubber dam (Kerr, USA). Straight-line access was established, and K-files (Dentsply Tulsa Dental, Oklahoma City, USA) were used to negotiate all the root canals.

**Figure 4:**
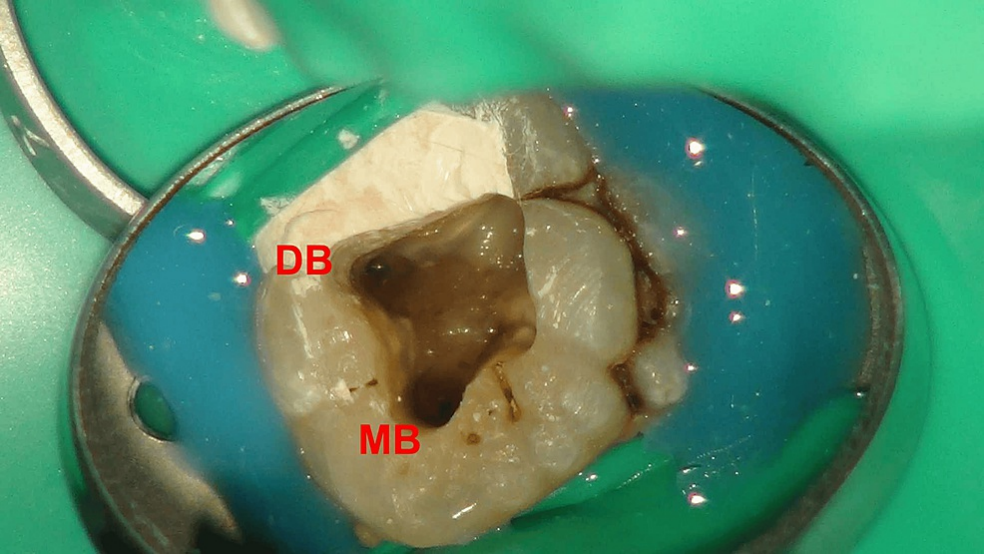
Microscopic photograph. The pulp floor is shown with the mesiobuccal (MB) and distobuccal (DB) root canal orifices.

**Figure 5:**
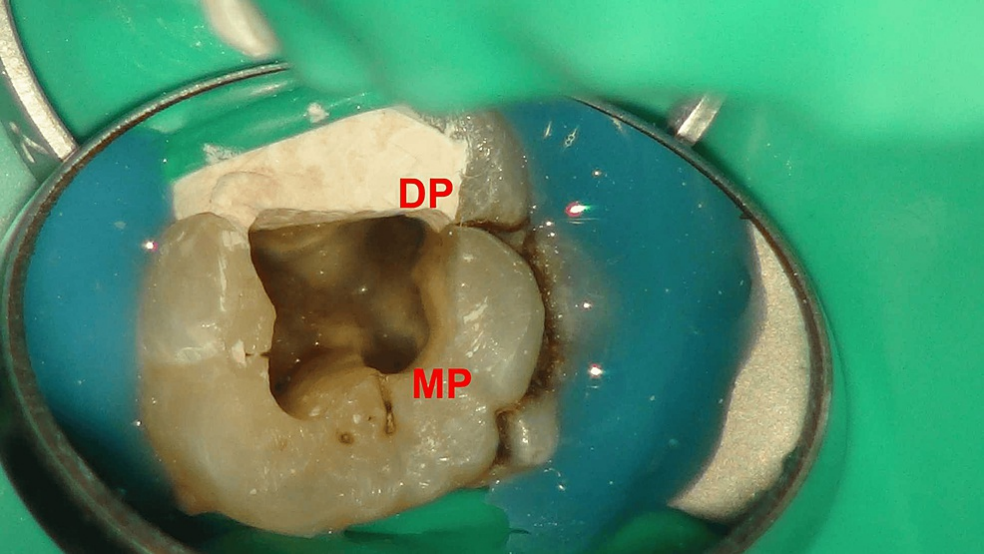
Microscopic photograph. The pulp floor is shown with two palatal root canal orifices, mesiopalatal(MP) and distopalatal (DP), being notably distant from each other.

**Figure 6:**
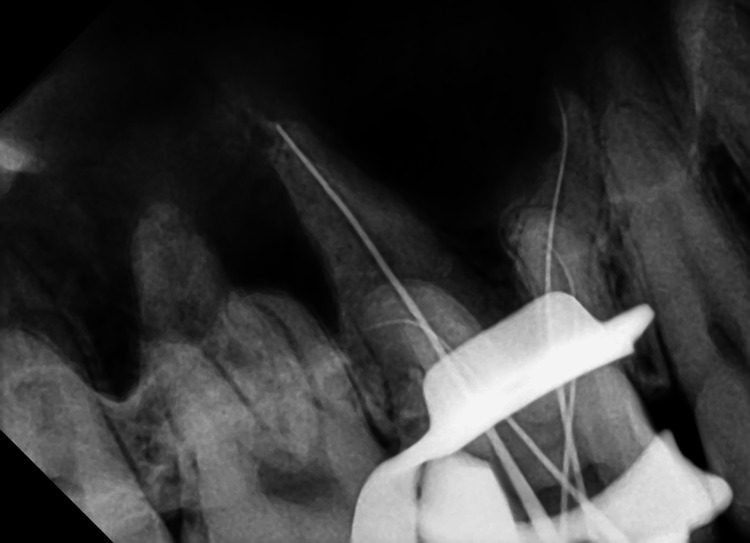
The periapical radiograph for working length confirmation. The working length of each canal is accurately determined by inserting a K-file into the canal and confirming its length.

Under the microscope, four root canal orifices in a trapezoidal arrangement were observed on the pulp floor, with the two palatal root canal orifices notably distant (Figures 4, 5). The root canal working lengths were measured with an electronic apex locator (J Morita Corp, Tokyo, Japan), and a periapical radiograph was taken to confirm the working length (Figure 6). The Twisted Files NiTi rotary system (TF; SybronEndo, Orange, USA) was used for shaping the canals, alternating irrigation with 3% sodium hypochlorite(NaOCl) solution and 17% ethylenediaminetetraacetic acid (EDTA) solution. The canals were soaked in 3% NaOCl solution, followed by three 20-second irrigation sessions using an Irri-Safe ultrasonic tip (Acteon, Merignac, France). Subsequently, the canals were irrigated with 17% EDTA solution, again employing the Irri-Safe ultrasonic tip for three separate 20-second sessions. After these treatments, the canals were thoroughly rinsed with sterile water and dried using paper points. Calcium hydroxide paste (Pulpdent™ paste; Pulpdent Corporation, Watertown, USA) was carefully introduced into the canals. Finally, the access cavity was securely sealed with a temporary filling material.

**Figure 7:**
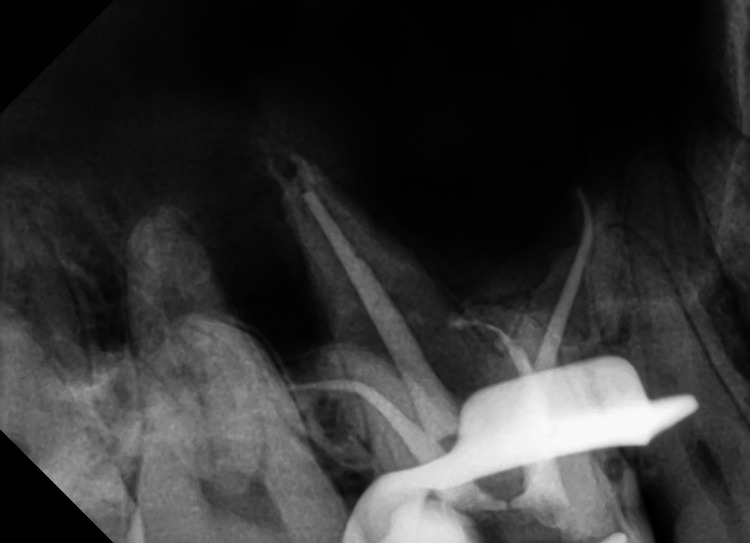
The postoperative radiograph of the upper right first molar. All four root canals are properly filled, with the two buccal canals exhibiting severe curvature.

One week later, the patient returned for follow-up. The temporary filling was removed, and the canals were rinsed with 3% NaOCl solution. The Irri-Safe ultrasonic tip removed the calcium hydroxide paste, followed by trial fitting of the main gutta-percha cones. The fit of the heat carrier tip (SybronEndo, Orange, USA), Buchanan hand pluggers (SybronEndo), and the needle of a 23G gutta-percha capsule (SybronEndo) was checked. The canals were irrigated following the same protocol as in the first visit. After the irrigation process, paper points were used to dry the canals, ensuring the removal of any residual moisture or leftover irrigation solution. The tip of the gutta-percha cones was dipped in a small amount of AH Plus sealer (Dentsply, Maillefer, Germany) before being placed in the canal. The root canal filling was completed using the continuous wave obturation technique with Elements Obturation Unit (SybronEndo), and a periapical radiograph was taken to evaluate the quality of root canal filling (Figure 7). The tooth restoration was completed with Filtek Z350 light-cured composite resin (3M ESPE, St. Paul, USA). The patient was advised to observe for one week before fully restoring crown. At 3-month follow-up examinations, the upper right first molar remained asymptomatic.

## Discussion

The anatomical variation in maxillary first molars is quite significant, with a single palatal root being more common, while the occurrence of two palatal roots is relatively rare [8,10]. Christie et al. have categorized maxillary molars with double palatal roots into three types [12]. In this case, the affected tooth closely resembles Type I of their classification, where the two palatal roots are long and widely spaced, and the two buccal roots are closer together, exhibiting severe curvature resembling cow horns [12].

Identifying and locating additional roots in such molars is crucial to avoid missing root canals during treatment. Practitioners must be well-versed in the various anomalies of tooth roots and root canals, including number and shape variations [2]. Careful observation and examination of the tooth's crown and cervical morphology are essential. Molars with extra roots often exhibit a bulbous crown appearance, additional cusps, and axial surface protrusions, with the tooth's cervical outline being palpable with a periodontal probe [19]. Compared to the contralateral left upper first molar, the affected tooth presented with a more pronounced Carabelli's cusp and a sixth cusp (Figure 3). To the best of the authors' knowledge, no published studies or case reports reveal any instances of an additional sixth cusp.

A thorough review of pre-operative X-rays is essential to identifying and locating additional roots [2]. Pre-operative X-ray imaging that clearly shows the periodontal ligament of the two palatal roots and their respective independent root canal images is the gold standard for diagnosis [12-14]. A blurred or unclear image of the palatal root or its canal contour could indicate the existence of a second palatal root. In such cases, taking additional mesial or distal offset radiographs is advisable, particularly for Type II configurations, where the buccal roots might overlap with the palatal root [12-14]. Utilization of special diagnostic methods, techniques, and equipment is sometimes necessary.

In some cases, pre-operative diagnosis, intraoperative navigation, and postoperative assessment might require CBCT [18]. Nevertheless, for patients with limited income or those who decline CBCT scans, capturing X-rays from various angles continues to be a cost-effective and realistic primary option. Clinically, to locate the second canal in the mesiobuccal root, the typical access opening for maxillary first molars is a mesially-tilted quadrilateral. For maxillary first molars with double palatal roots, especially those corresponding to Christie's Type I, the mesiodistal diameter is wider than the lingual tip, necessitating a larger than usual access opening to expose the extra root canal orifices [12].

In cases where the tooth's pulp chamber is calcified or secondary dentin blocks the root canal orifice, the blockage needs to be removed under a dental operating microscope using ultrasonic tips, combined with methods like the champagne bubble test, methylene blue dye, and fiber optic transillumination for locating the root canal orifice [2,16]. If necessary, analysis of limited field-of-view CBCT images for three-dimensional localization of the root canal orifice can be performed, calculating the distance between the blocked orifice and reference points and the thickness of the obstruction [16,18]. This approach allows for more precise operations, minimizing damage to the tooth structure and preventing perforation of the pulp chamber floor or root canal. However, the "as low as reasonably achievable (ALARA)" principle should always be considered to ensure patient safety.

## Conclusions

Successfully treating maxillary first molars with complex root variations, especially those with additional palatal roots, demands a comprehensive understanding of root anatomy, meticulous pre-operative planning, and the use of advanced diagnostic and surgical techniques to ensure accurate identification and treatment of all root canals, thereby enhancing treatment outcomes.
